# Metabolomics reveals potential biomarkers in the rumen fluid of dairy cows with different levels of milk production

**DOI:** 10.5713/ajas.19.0214

**Published:** 2019-08-23

**Authors:** Hua Zhang, Jinjin Tong, Yonghong Zhang, Benhai Xiong, Linshu Jiang

**Affiliations:** 1 Beijing Key Laboratory for Dairy Cow Nutrition, Beijing University of Agriculture, Beijing, 102206, China; 2 Beijing Bei Nong Enterprise Management Co., Ltd., Beijing 102206, China; 3 State Key Laboratory of Animal Nutrition, Institute of Animal Science, Chinese Academy of Agricultural Sciences, Beijing 100193, China

**Keywords:** Metabolomics, High/low Milk Production, Rumen Metabolism, Dairy Cows

## Abstract

**Objective:**

In the present study, an liquid chromatography/mass spectrometry (LC/MS) metabolomics approach was performed to investigate potential biomarkers of milk production in high- and low-milk-yield dairy cows and to establish correlations among rumen fluid metabolites.

**Methods:**

Sixteen lactating dairy cows with similar parity and days in milk were divided into high-yield (HY) and low-yield (LY) groups based on milk yield. On day 21, rumen fluid metabolites were quantified applying LC/MS.

**Results:**

The principal component analysis and orthogonal correction partial least squares discriminant analysis showed significantly separated clusters of the ruminal metabolite profiles of HY and LY groups. Compared with HY group, a total of 24 ruminal metabolites were significantly greater in LY group, such as 3-hydroxyanthranilic acid, carboxylic acids, carboxylic acid derivatives (L-isoleucine, L-valine, L-tyrosine, etc.), diazines (uracil, thymine, cytosine), and palmitic acid, while the concentrations of 30 metabolites were dramatically decreased in LY group compared to HY group, included gentisic acid, caprylic acid, and myristic acid. The metabolite enrichment analysis indicated that protein digestion and absorption, ABC transporters and unsaturated fatty acid biosynthesis were significantly different between the two groups. Correlation analysis between the ruminal microbiome and metabolites revealed that certain typical metabolites were exceedingly associated with definite ruminal bacteria; *Firmicutes*, *Actinobacteria*, and *Synergistetes* phyla were highly correlated with most metabolites.

**Conclusion:**

These findings revealed that the ruminal metabolite profiles were significantly different between HY and LY groups, and these results may provide novel insights to evaluate biomarkers for a better feed digestion and may reveal the potential mechanism underlying the difference in milk yield in dairy cows.

## INTRODUCTION

Nutritional requirements plays an important role in dairy cows’ productivity, it is necessary to find an effectively digested feed and to maintain healthy physiological functions for efficient milk production. Interestingly, our previous study revealed that the compositions and structures of ruminal bacterial communities between high-yield (HY) and low-yield (LY) dairy cows were significantly different, which advanced the understanding underlying regulation response to the different milk yield [1]. Although the rumen microbial population has an explicit relationship with volatile fatty acids (VFAs), amino acids as well as fatty acids, ruminal metabolites can comprehensively reflect rumen digestive and systemic health [2]. However, the nutrient metabolism in the ruminal system between dairy cows having different ability in milk production remains unclear. Therefore, further research exploring the metabolic profile is necessary to elucidate the mechanism of metabolic regulation of dairy cows.

Metabolomics is an innovative global assessment and quantitatively measures the small endogenous metabolites in biological sample using high-throughput techniques, such as nuclear magnetic resonance [3], liquid chromatography/mass spectrometry (LC/MS) [4], gas chromatography–mass spectrometry [5] and gas chromatography time-of-flight/mass spectrometry (GC-TOF/MS) [6]; moreover, in recent years, metabolomics approaches have been widely utilized for their high resolution and detection sensitivity for ruminal metabolite biomarkers. Most previous studies employed interesting metabolomics applications to reveal the impact of diet on animal health and performance [7], to investigate potential metabolite biomarkers of disease or physiology [4,8] and to examine diet-induced metabolic alterations in the ruminal microbial metabolic profile and their effects on milk production-related traits in ruminants [5,9,10]. Thus, the identification and integrative analysis of ruminal metabolites may facilitate the comprehensive characterization of metabolic mechanisms at the molecular and cellular levels in response to internal or external stimuli.

Therefore, it is interesting whether differences in the milk yields of cows were directly associated with their ruminal bacterial metabolomics. The objective of this study was to illustrate the metabolic mechanisms underlying the differences between high and low milk production, especially the rumen bacterial community, by examining the metabolite profiles of ruminal fluid in dairy cows.

## MATERIALS AND METHODS

### Animals and experimental design

The experimental procedures were approved by the Animal Care Committee, Beijing University of Agriculture (Beijing, China). The experiment was designed as previously described [1]. Briefly, sixteen Holstein lactating dairy cows were divided into two groups according to the characteristics of parity (2.6 ±0.4), average body weight (670±24 kg), average dry mater intake (24.2±2.7 kg/d), number of lactation days (114.6±7.5), and these cows were assigned to either HY or LY group (31.90 ±1.76 kg/d and 19.30±1.76 kg/d, respectively; n = 8). The experiment was conducted over 21 days, with the first 14 days serving as an adaptation period. Cows were individually housed in tethered stalls in a barn with good ventilation and were fed and milked using a pipeline milking system three times per day at 0630, 1400, and 2000 h. The methods of samples collection and analysis were described in our previous study [1].

### Liquid chromatography/mass spectrometry analysis

The rumen fluid samples were thawed at room temperature. Samples (100 μL) were used for metabolomics analysis. L-2-chlorophenylalanine (10 μL, 0.3 mg/mL), added, mixed and shaken for 10 s. Then, 300 μL of methanol-acetonitrile (2:1, v/v) added, mixed and shaken for 60 s. The solution was ultrasonically extracted on ice for 5 min, incubated at −20°C for 30 min and then centrifuged for 15 min at 3,850 g at 4°C. Two hundred microliters of supernatant was used for LC/MS analysis.

The LC/MS was carried out using an Ultimate 3000-Velos Pro system equipped with a binary solvent delivery manager and a sample manager, coupled with an LTQ Orbitrap mass spectrometer equipped with an electrospray interface (Thermo Fisher Scientific, Sunnyvale, CA, USA). The LC was an Acquity BEH C18 column (100 mm×2.1 mm i.d., 1.7 μm; Waters, Milford, MA, USA). Separation was achieved with solvent B (acetonitrile) and solvent A (aqueous 0.1% (v/v) formic acid) with the following gradient at a flow rate of 0.40 mL/min: 5% B–25% B over 0–1.5 min; 25% B–100% B over 1.5–10.0 min; 100% B–100% B over 10.0–13.0 min; 100% B–5% B over 13.0–13.5 min; and 13.5–14.5 min holding at 5% B. The injection volume was 3.0 μL, and the column temperature was set at 45.0°C. The mass spectrometric data were collected using an LTQ Orbitrap mass spectrometer equipped with an electrospray ionization source operating in either positive or negative ion mode. The capillary and source temperatures were set at 350°C, with a desolvation gas flow of 45 L/h. Centroid data were collected from 50 to 1,000 m/z with a 30,000 resolution.

The quality control (QC) sample was prepared by mixing aliquots of all samples into a pooled sample. Then, the QC sample was analysed using the same method as the experimental samples. The QC samples were injected at regular intervals (every 10 samples) throughout the analytical run to provide a set of data to assess repeatability.

### Statistical analysis

The positive and negative data were combined to obtain a combined dataset that was imported into the SIMCA-P+ 14.0 software package (Umetrics, Umeå, Sweden). Principle component analysis (PCA) [11] and (orthogonal) partial least squares discriminant analysis ((O)PLS-DA) were carried out to visualize the metabolic alterations among experimental groups after mean centring and unit variance scaling. The variable importance in the projection (VIP) ranks the overall contribution of each variable to the (O)PLS-DA model, and those variables with VIP>1.0 are considered relevant for group discrimination. In this study, the default 7-round cross-validation was applied, and one-seventh of the samples were excluded from the mathematical model in each round to guard against overfitting. Significant differences in the metabolites of the two groups were analysed using the Wilcoxon rank-sum test. A heatmap of the key metabolites analyze in this process with methods similar to those previously published [12]. The correlation matrix between metabolites and ruminal bacterial species was generated using Spearman correlation coefficient and visualized by using R language.

## RESULTS

### Ruminal pH, volatile fatty acids, dry matter intake, milk yield and milk composition

Data describing the ruminal parameters and performance of the experimental cows have been reported in Tong’s prior study [1]. Briefly, the ruminal pH was similar in both HY and LY groups. Compared with LY group, the proportion of propionate (p = 0.08) and total VFA concentration (p<0.05) in HY group were increased, whereas the acetate to propionate ratio (p = 0.06) and the proportion of acetate (p = 0.06) in HY group showed decreasing trends. In addition, the dry matter intake was higher in HY group than LY group (p = 0.03). Compared with LY group, milk production, 4% fat-corrected milk yield and energy-corrected milk yield were significantly increased in HY group. However, there was an increasing trend in milk fat content (p = 0.08) and milk protein content (p<0.01) in LY group compared to HY group. No differences were observed in milk lactose content and somatic cell count between LY and HY groups.

### Differences in the ruminal metabolites of the two groups

In total, 10,544 practicable peaks that were unique and non-overlapping were obtained from the rumen fluid samples. After strict QC and identification, 366 metabolites were differentially detected in LY and HY groups. These metabolites were mainly glycerophosphoethanolamines, fatty acids, amino acids, peptides, carbohydrates and pyrimidines.

**Figure 1:**
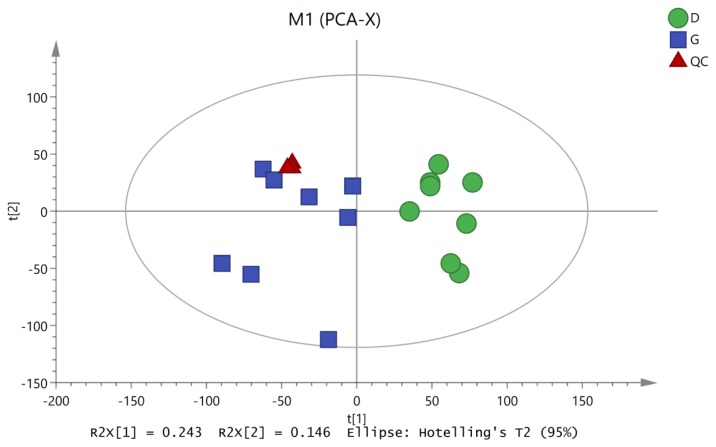
Principal component analysis (PCA) of the liquid chromatography/mass spectrometry metabolite profiles of the quality control (QC), high-yield and low-yield dairy cow rumen fluid samples. The green circles represent low-yield cows, the blue squares represent high-yield cows, and the red triangles represent the QC samples.

**Figure 2:**
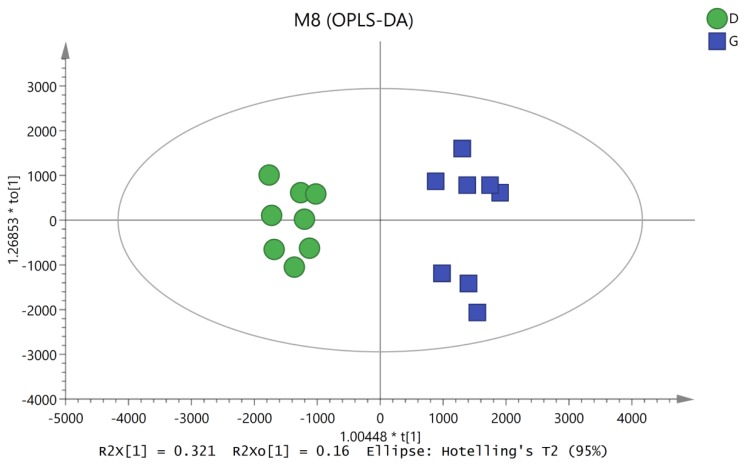
Orthogonal correction partial least squares discriminant analysis (OPLS-DA) derived from the liquid chromatography/mass spectrometry metabolite profiles of the ruminal fluid samples from high-yield (HY) and low-yield (LY) group dairy cows. The green circles represent LY cows, and the blue squares represent HY cows.

To characterize the variations in the metabolic profiles of HY and LY dairy cows, PCA and OPLS-DA were conducted. As presented on the PCA score plots (Figure 1), HY and LY groups were clearly separated; PCA 1 and PCA 2 accounted for 25.8% and 15.9% of the total variation, respectively. Furthermore, in our study, the PCA of the LC/MS data showed that all the QC samples obtained in the positive and negative ion modes mostly overlapped in the same area, which indicated that this model was stable, reproducible and consistent for all the samples. As shown in Figure 2, not surprisingly, OPLS-DA model presented that the rumen fluids of HY and LY dairy cows had significantly different and distinct metabolite compositions. Both PCA and OPLS-DA indicated that metabolites found in the rumen fluid samples of HY and LY dairy cows were markedly distinct.

**Figure 3:**
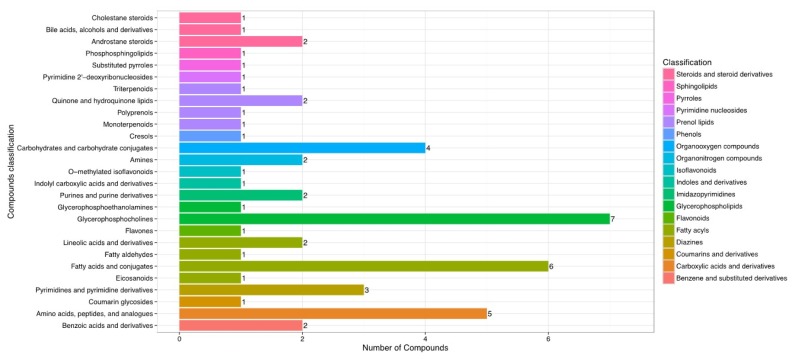
Different metabolite contents in the ruminal fluid between high-yield and low-yield group dairy cows.

After the results of statistical analysis and the VIP value obtained from OPLS-DA were considered, 54 significantly different metabolites (p<0.05 and VIP>1) between the rumen fluids of HY and LY dairy cows were identified, as shown in Table 1. These identified metabolites were diverse and included the following: benzene and substituted derivatives; carboxylic acids and derivatives; coumarins and derivatives; diazines; fatty acyls; glycerophospholipids; indoles and derivatives; organooxygen compounds; pyrimidine nucleosides; steroids and steroid derivatives. As shown in Figure 3, each class of compound had multiple differentially abundant metabolites in the rumen fluids of HY and LY dairy cows.

Compared with HY group, 24 ruminal metabolites were significantly increased in LY group, such as 3-hydroxyanthranilic acid, carboxylic acids, carboxylic acid derivatives (L-isoleucine, L-valine, L-tyrosine, L-lysine, dimethylglycine), diazines (uracil, thymine, cytosine), palmitic acid, and imidazopyrimidines. The remaining 30 metabolites were decreased in LY group, such as gentisic acid, caprylic acid, myristic acid, linoleic acid, and glycerophospholipids (p<0.05). Interestingly, remarkable alterations in the content of carboxylic acids, carboxylic acid derivatives, coumarins, coumarin derivatives, diazines, imidazopyridines, indoles, indole derivatives, organonitrogen compounds, phenols, and pyrroles in LY and HY groups were observed in the present study. Specifically, the pyrrole metabolites and fluvastatin levels were increased by 6.06-fold (p<0.01) in LY group compared with HY group.

**Figure 4:**
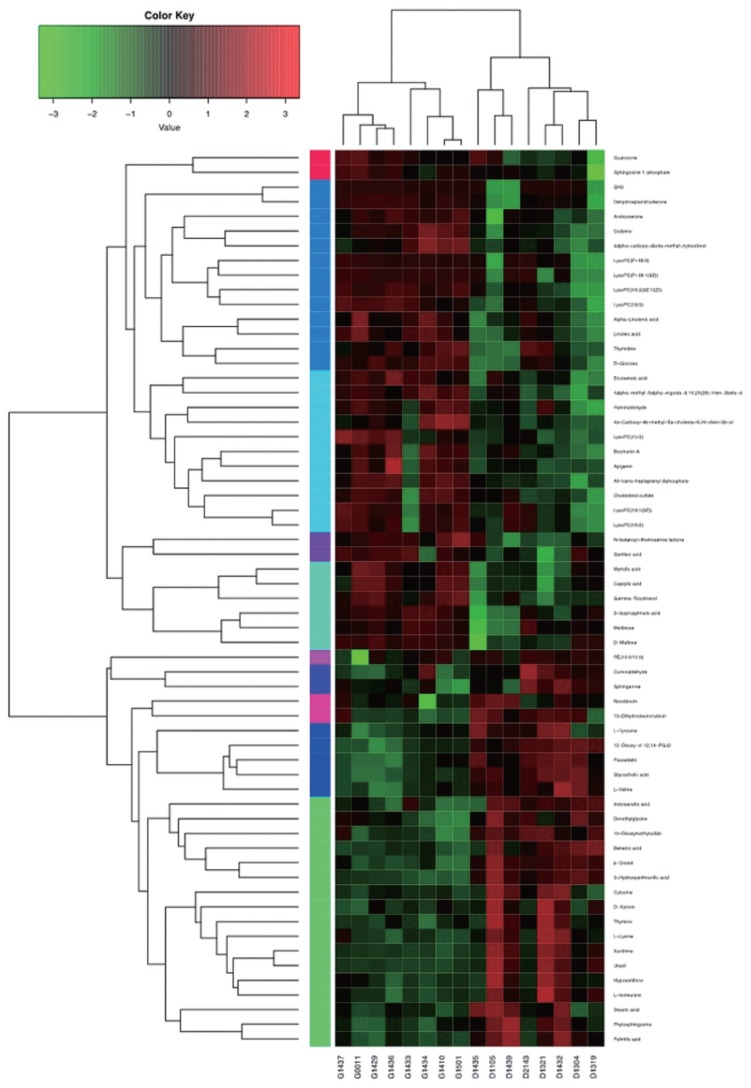
Hierarchical clustering analysis for identified the different metabolites from the ruminal fluid of high-yield and low-yield dairy cows. Cells were coloured based on the signal intensity detected in the ruminal samples: light red squares represent high content in the rumen; green squares represent low content and black represents intermediate levels.

As shown in Figure 4, hierarchical clustering analysis (HCA) shows significantly changed metabolite responses to different milk yields in dairy cows, which provides a further understanding of the metabolites that differ between HY and LY dairy cows. In the present study, HCA revealed that the significantly increased metabolites in LY group compared with HY group were mainly gathered into two subclusters. As shown in the lower part of Figure 4, compared with HY group, the metabolites either increased or decreased in LY group were separated clearly and significantly different subclusters were located. One subcluster consisted of the significantly upregulated metabolites in LY group compared with HY group, such as 3-hydroxyanthranilic acid, L-lysine, thymine and uracil. The other subcluster consisted of the remarkably upregulated metabolites from HY group, including glycerophospholipids, prenol lipids, and organooxygen compounds.

### Metabolic pathway analyses

To further understand how multiple pathways alternated in response to HY and LY milk production, analysis of the functions of the pathways association with differential metabolites was subjected to the Kyoto encyclopedia of genes and genomes (KEGG). The dramatically impacted pathways identified with this enrichment analysis are shown in Table 2. ABC transporters, 2-oxocarboxylic acid metabolism, biosynthesis of amino acids, biosynthesis of unsaturated fatty acids and protein digestion and absorption were determined to be the main pathways that were different between HY and LY in the present study.

**Figure 5:**
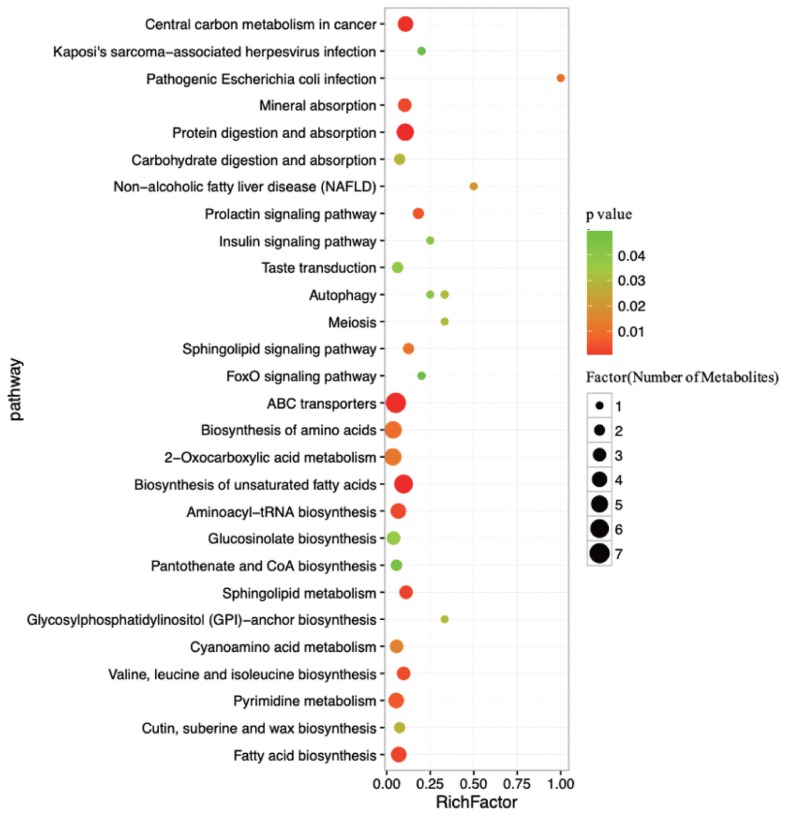
Pathways associated with the metabolites identified in both the high- and low-milk-yield dairy cows. The x-axis represents the pathway richness factor, and the y-axis represents the pathway name. Large sizes and dark colours represent a large number of metabolites and high pathway impact values, respectively.

As determined by pathway topology analysis and as shown in Figure 5, 13 main metabolic pathways were enriched in our study, including the followings: ABC transporters; biosynthesis of unsaturated fatty acids; protein digestion and absorption; 2-oxocarboxylic acid metabolism; biosynthesis of amino acids; fatty acid biosynthesis; aminoacyl-tRNA biosynthesis; pyrimidine metabolism; cyanoacetic acid metabolism; glucose-inolate biosynthesis; sphingolipid metabolism; valine, leucine and isoleucine biosynthesis; and mineral absorption. Among these metabolic pathways, protein digestion and absorption, ABC transporters and biosynthesis of unsaturated fatty acids were the three most impacted pathways between the two groups, as determined by the richness factor values.

### Correlation analysis between the ruminal microbiome and metabolites

**Figure 6:**
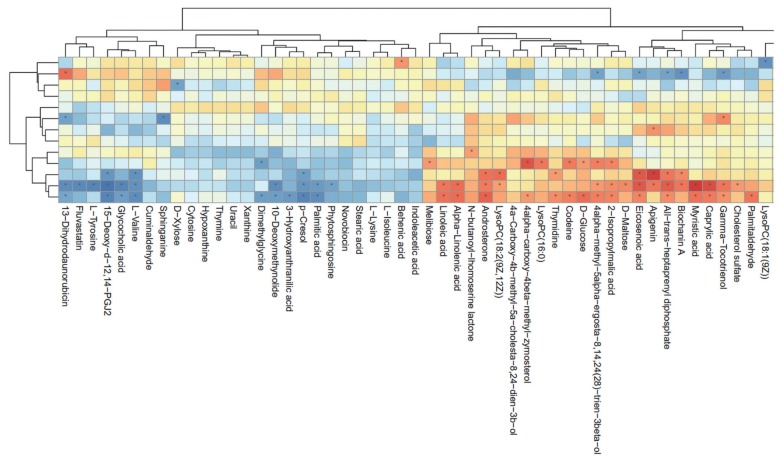
Correlation plot showing the correlation between the perturbed ruminal bacteria phyla and altered ruminal metabolites (* p<0.05).

For further understanding of the functional correlation between the perturbations in the ruminal microbiome and metabolites as a result of different levels of milk production, Spearman correlation coefficient analysis was conducted in the present study. Clear correlations were identified between the perturbed ruminal microbiome and altered metabolite profiles (r>0.5 or <−0.5, p<0.05). Figure 6 shows that several definite metabolites were greatly correlated with specific ruminal bacteria, which established the functional correlation between the microbiome and metabolites in rumen. It was found that *Firmicutes*, *Actinobacteria*, and *Synergistetes* phyla were highly correlated with most metabolites. Additionally, *Bacteroidetes* phylum was significantly correlated with only D-xylose, *Saccharibacteria* was significantly correlated with only apigenin, and *Fibrobacteres* was significantly correlated with only N-butanoyl-homoserine lactone.

## DISCUSSION

In the present study, LC/MS metabolomics was used to evaluate the metabolites in the ruminal fluid of HY and LY dairy cows. We also wanted to investigate whether the observed changes in metabolites could provide further insight into specific ruminal microflora-related changes and aimed to provide a new perspective regarding perturbations of ruminal metabolism to reveal the mechanism of ruminal digestion in dairy cows with different milk yields.

Our results showed a clear separation of the ruminal metabolites in HY and LY dairy cows, indicating significant differences in metabolic composition. In the present study, L-isoleucine, L-valine, L-tyrosine, L-lysine, and dimethylglycine were significantly increased in LY group than HY group. Allison [13] previously reported that valine is generated from the main substrates pyruvate and isobutyrate. In addition, pyruvate can be converted to lactate by lactate dehydrogenase [14] or to acetyl coenzyme-A and formate by pyruvate formate-lyase [15], then acetyl-CoA is converted either to acetate or ethanol. Moreover, Andries et al [16] reported that branched chain fatty acids are also synthesized from deamination of the amino acids valine, isoleucine, leucine, and proline. Our previous study showed that acetate was remarkably increased in LY group compared with HY group, which further suggested increased substrate for amino acid synthesis. Furthermore, the enriched pathways observed in the present study, including aminoacyl-tRNA biosynthesis, amino acid biosynthesis, cyanoamino acid metabolism, glucosinolate biosynthesis, pantothenate and CoA biosynthesis as well as protein digestion and absorption, also confirmed the above hypothesis. Thus, we can reasonably speculate that high levels of acetate could promote the biosynthesis of amino acids in the rumen, and thereby this metabolite could reflect the condition of digestion in the rumen.

Moreover, compared with LY group, myristic acid, palmitaldehyde, linoleic acid, and alpha-linolenic acid were significantly increased in HY group. The concentrations of ruminal long-chain fatty acids are indicative of active lipolysis, biohydrogenation and microbial fatty acid synthesis in the rumen [17]. Additionally, before these unsaturated fatty acids can be rapidly hydrogenated by microbes into saturated end products, lipases, galactosidases, and phospholipases produced by ruminal microbes remove nonesterified fatty acids [18]. Furthermore, palmitic acid, linoleic acid and alpha-linolenic acid were reported could be as sensitive and specific candidate biomarkers to distinguish low- and high-quality steers [19]. Therefore, it could be concluded that these fatty acids were detected at relatively high levels due to the microbial fermentation in the rumen of HY group; microbial fermentation and the efficient absorption of violate fatty acids may explain the mechanism of different milk yields.

One remarkable alteration observed in this study were the increased levels of D-maltose and D-glucose in HY group compared with LY group. The nutrients in the diet could be degraded to maltose by amylase and then to glucose by maltase or maltose phosphorylase in the rumen. Previous research has also indicated that high levels of glucose promote the production of pyruvate in the rumen [20,21]. Glucose is the major monosaccharide liberated during the degradation of starch, and it can be converted into a number of different polyols and amino acids via the glycolytic pathway and its branches [22]. Microorganisms convert carbohydrates to pyruvate and acetyl-CoA by glycolytic pathway and pentose phosphate pathway [14]. Furthermore, previous studies demonstrated that carbohydrate metabolism caused significant increases in lactate and propionate concentration as well as decline in the acetate concentration [8,23], which was in accordance with our previous results that acetate levels were lower and propionate levels were greater in HY dairy cows relative to the LY dairy cows. It has also been reported that diets rich in readily available carbohydrates are associated with alterations in the rumen microbiota, which are followed by significant changes in the metabolic pathways of the rumen [8,24]. Interestingly, our correlation results revealed that D-glucose was significantly positively correlated with *Firmicutes* and that D-maltose was significantly positively correlated with *Actinobacteria*. Based on our integrated pathway analysis, it is reasonable to conclude that D-maltose and D-glucose metabolites were increased in HY group compared with LY group.

One of the most interesting observations from this study was that phyla of the ruminal microbiome were specifically correlated with the different metabolites detected in HY and LY dairy cows. It is well known that ruminal metabolites do not independently exist, and a comprehensive understanding of the variation in other metabolites is warranted. In the present study, *Firmicutes* was mostly correlated with fatty acyls. Furthermore, it has been reported that *Firmicutes* plays an important role in milk production [25]. Thus, it could be suggested that the fatty acyl metabolites contributed to the increased abundance of these bacteria in the rumen fluid. Therefore, these correlations of ruminal metabolites with the ruminal microbiome can reflect metabolic processes. Additionally, *Bacteroidetes* was significantly negatively correlated with only D-xylose, and D-xylose level was significantly increased in HY group compared with LY group. Accumulated evidence strongly suggests that the metabolic alterations associated with microbiome perturbations are important biomarkers that indicate the health [19], nutrition [26], or dietary changes in response to the physiology [27] of dairy cows. Taken together, these results indicate that rumen microbes promote protein degradation in HY dairy cows, which provides a better understanding of the difference in the milk proteins of the two groups. Therefore, correlations between ruminal metabolites and ruminal microbiome in the present study may provide new information for advanced understanding of complex rumen metabolism.

## CONCLUSION

These data indicated that not only the metabolites in the ruminal fluids were significantly different between HY and LY cows, but also these metabolites associated with the ruminal microbiota affected the metabolic function. These highly correlated metabolites may be potential biomarkers for the measurement of digestive and rumen function. In this study, the integration of the high-throughput sequencing of the ruminal microbiome and metabolomics should provide new insight to improve the understanding of the physiological and metabolic mechanisms involved in milk production. Moreover, these findings suggest that due to host–microbiota interactions, dairy cows with different milk yields have different metabolite content, which are most likely correspond to definite differences between ruminal fermentation and milk component.

## Figures and Tables

**Table 1:** Differential metabolites in the ruminal fluid of high-yield and low-yield group dairy cows

Metabolites	Compound ID	Formula	m/z	Retention time (min)	VIP value	FC1) (D/G)	p-value
Benzene and substituted derivatives							
3-Hydroxyanthranilic acid	HMDB01476	C7H7NO3	171.08	10.42	7.13	2.21	0.00
Gentisic acid	HMDB0000152	C7H6O4	153.02	3.12	2.72	0.80	0.02
Organic acids and derivatives							
L-Isoleucine	HMDB00172	C6H13NO2	132.10	1.05	7.81	1.44	0.01
L-Valine	HMDB00883	C5H11NO2	100.08	1.50	4.32	1.53	0.00
L-Tyrosine	HMDB00158	C9H11NO3	164.07	3.62	2.87	1.82	0.01
L-Lysine	HMDB00182	C6H14N2O2	147.11	0.57	2.18	1.98	0.01
Dimethylglycine	HMDB00092	C4H9NO2	86.06	1.02	1.45	1.34	0.01
Coumarins and derivatives							
Novobiocin	HMDB0015185	C31H36N2O11	593.22	4.72	1.09	1.89	0.04
Diazines, pyridine							
Uracil	HMDB00300	C4H4N2O2	113.03	0.80	2.66	1.83	0.00
Thymine	HMDB00262	C5H6N2O2	127.05	1.00	2.53	1.36	0.01
Cytosine	HMDB00630	C4H5N3O	112.05	0.65	1.27	1.84	0.03
Fatty acyls							
15-Deoxy-d-12,14-PGJ2	HMDB05079	C20H28O3	334.24	4.72	1.22	1.71	0.00
Palmitic acid	HMDB00220	C16H32O2	274.27	5.86	10.94	1.44	0.00
Behenic acid	HMDB00944	C22H44O2	358.37	13.16	4.22	2.70	0.00
Stearic acid	HMDB00827	C18H36O2	302.31	7.03	2.42	1.24	0.02
Caprylic acid	HMDB0000482	C8H16O2	287.22	5.54	1.17	0.35	0.00
2-Isopropylmalic acid	HMDB0000402	C7H12O5	221.07	0.65	1.11	0.43	0.00
Myristic acid	HMDB0000806	C14H28O2	273.21	5.37	1.06	0.13	0.02
Palmitaldehyde	HMDB0001551	C16H32O	285.24	9.04	2.30	0.58	0.01
Linoleic acid	HMDB00673	C18H32O2	561.49	9.70	3.71	0.29	0.00
Alpha-Linolenic acid	HMDB01388	C18H30O2	279.23	8.86	1.75	0.57	0.01
Flavonoids							
Apigenin	HMDB0002124	C15H10O5	269.05	4.72	1.57	0.33	0.03
Glycerophospholipids							
LysoPC (16:0)	HMDB0010382	C24H50NO7P	540.33	7.34	4.80	0.43	0.02
LysoPC (15:0)	HMDB0010381	C23H48NO7P	480.31	8.56	4.32	0.39	0.01
LysoPC (18:0)	HMDB10384	C26H54NO7P	524.37	8.50	3.69	0.18	0.00
LysoPC (18:1(9Z))	HMDB02815	C26H52NO7P	522.36	7.28	3.19	0.10	0.00
LysoPC (18:2(9Z,12Z))	HMDB10386	C26H50NO7P	520.34	6.57	2.54	0.17	0.00
LysoPC (P-18:1(9Z))	HMDB10408	C26H52NO6P	506.36	7.81	2.32	0.21	0.00
LysoPC (P-16:0)	HMDB10407	C24H50NO6P	480.34	7.54	2.21	0.26	0.00
Phosphatidylethanolamine (PE; 14:0/14:0)	HMDB0008821	C33H66NO8P	634.44	11.00	2.31	2.93	0.03
Imidazopyrimidines							
Hypoxanthine	HMDB00157	C5H4N4O	137.05	0.82	6.02	1.48	0.03
Xanthine	HMDB00292	C5H4N4O2	153.04	0.83	2.44	1.77	0.00
Indoles and derivatives							
Indoleacetic acid	HMDB0000197	C10H9NO2	220.06	3.82	1.14	3.14	0.00
Isoflavonoids							
Biochanin A	HMDB0002338	C16H12O5	329.07	4.78	1.39	0.52	0.01
Organonitrogen compounds							
Phytosphingosine	LMSP01030001	C18H39NO3	318.30	5.90	9.58	1.43	0.01
Sphinganine	HMDB00269	C18H39NO2	284.29	10.46	3.50	1.27	0.04
Organooxygen compounds							
D-Maltose	HMDB00163	C12H22O11	365.11	0.63	5.23	0.56	0.00
D-Glucose	HMDB0000122	C6H12O6	225.06	0.66	4.60	0.52	0.00
Melibiose	HMDB0000048	C12H22O11	387.11	0.66	2.55	0.59	0.03
D-Xylose	HMDB00098	C5H10O5	173.04	0.63	1.02	1.54	0.04
Phenols							
p-Cresol	HMDB01858	C7H8O	91.05	2.37	3.37	1.51	0.00
Prenol lipids							
Cuminaldehyde	HMDB0002214	C10H12O	193.09	5.32	1.22	1.43	0.03
All-trans-heptaprenyl diphosphate	HMDB0012187	C35H60O7P2	699.38	7.35	2.36	0.02	0.00
Gamma-tocotrienol	HMDB0012958	C28H42O2	455.32	6.66	3.41	0.37	0.00
QH2	HMDB01304	C14H2OO4.[C5H8]n	321.21	7.35	1.44	0.43	0.00
4a-Carboxy-4b-methyl-5a-cholesta-8,24-dien-3b-ol	HMDB0001181	C29H46O3	441.34	8.08	3.44	0.34	0.03
Pyrimidine nucleosides							
Thymidine	HMDB0000273	C10H14N2O5	287.09	0.92	1.70	0.63	0.05
Pyrroles							
Fluvastatin	HMDB0015227	C24H26FNO4	392.17	6.33	1.37	6.06	0.00
Sphingolipids							
Sphingosine 1-phosphate	HMDB0000277	C18H38NO5P	378.24	6.01	1.02	0.11	0.04
Steroids and steroid derivatives							
Dehydroepiandrosterone	HMDB0000077	C19H28O2	333.21	7.87	3.15	0.26	0.00
Androsterone	HMDB00031	C19H30O2	603.44	13.82	1.13	0.41	0.00
Glycocholic acid	HMDB00138	C26H43NO6	448.31	4.89	1.72	1.84	0.00
Cholesterol sulfate	HMDB0000653	C27H46O4S	465.30	10.63	3.17	0.54	0.01

VIP, variable importance in the projection.

1) FC represents the fold change, the ratio of the mean value of the peak area obtained from low-yield cows and the mean value of the peak area obtained from high-yield cows. FC>1 indicates that this metabolite is more abundant in the low-yield cows than in the high-yield cows.

**Table 2:** Metabolic pathways of the significantly enriched metabolites between high-yield and low-yield dairy cows

Metabolic pathways	Class	Metabolites	p-value
Autophagy	Cellular processes (1)	PE (14:0/14:0)	0.030
Meiosis	Cellular processes (1)	D-Glucose	0.030
ABC transporters	Environmental information processing (7)	D-Glucose; L-Isoleucine; L-Lysine; D-Maltose; Melibiose; L-Valine; D-Xylose;	0.000
FoxO signalling pathway	Environmental information processing (1)	D-Glucose;	0.049
Sphingolipid signalling pathway	Environmental information processing (2)	Sphinganine; Sphingosine 1-phosphate	0.011
Aminoacyl-tRNA biosynthesis	Genetic information processing (4)	L-Lysine; L-Isoleucine; L-Tyrosine; L-Valine	0.003
2-Oxocarboxylic acid metabolism	Metabolism (5)	2-Isopropylmalic acid; L-Isoleucine; L-Tyrosine; L-Lysine; L-Valine	0.012
Biosynthesis of amino acids	Metabolism (5)	2-Isopropylmalic acid; L-Isoleucine; L-Tyrosine; L-Lysine; L-Valine	0.010
Biosynthesis of unsaturated fatty acids	Metabolism (6)	Alpha-linolenic acid; Palmitic acid; Linoleic acid; Eicosenoic acid; Stearic acid; Behenic acid	0.000
Cutin, suberine and wax biosynthesis	Metabolism (2)	Palmitic acid; Behenic acid	0.029
Cyanoamino acid metabolism	Metabolism (3)	L-Isoleucine; L-Tyrosine; L-Valine	0.015
Fatty acid biosynthesis	Metabolism (4)	Stearic acid; Palmitic acid; Myristic acid; Caprylic acid	0.003
Glucosinolate biosynthesis	Metabolism (3)	L-Isoleucine; L-Tyrosine; L-Valine	0.037
Glycosylphosphatidylinositol (GPI)-anchor biosynthesis	Metabolism (1)	PE (14:0/14:0)	0.030
Pantothenate and CoA biosynthesis	Metabolism (2)	Uracil; L-Valine	0.047
Pyrimidine metabolism	Metabolism (4)	Uracil; Thymidine; Thymine; Cytosine	0.006
Sphingolipid metabolism	Metabolism (3)	Sphinganine; Phytosphingosine; Sphingosine 1-phosphate	0.002
Valine, leucine and isoleucine biosynthesis	Metabolism (3)	2-Isopropylmalic acid; L-Isoleucine; L-Valine	0.004
Carbohydrate digestion and absorption	Organismal systems (2)	D-Maltose; D-Glucose	0.029
Insulin signalling pathway	Organismal systems (1)	D-Glucose	0.040
Mineral absorption	Organismal systems (3)	L-Isoleucine; L-Valine; D-Glucose	0.003
Prolactin signalling pathway	Organismal systems (2)	L-Tyrosine; D-Glucose	0.005
Protein digestion and absorption	Organismal systems (5)	L-Lysine; L-Isoleucine; L-Tyrosine; p-Cresol; L-Valine	0.000
Taste transduction	Organismal systems (2)	D-Maltose; D-Glucose	0.038

The numbers in parentheses represent the number of metabolites enriched in the corresponding pathways.
